# Evaluation of risk correlation between recurrence of patellar dislocation and damage to the medial patellofemoral ligament in different sites caused by primary patellar dislocation by MRI: a meta-analysis

**DOI:** 10.1186/s13018-020-01984-0

**Published:** 2020-10-07

**Authors:** Boyong Jiang, Chenggang Qiao, Yuting Shi, Yizhong Ren, Changxu Han, Yong Zhu, Yuyan Na

**Affiliations:** 1 Baotou Medical College, Inner Mongolia University of Science and Technology, Jiuyuan District, Baotou, 014060 Inner Mongolia Autonomous Region China; 2 Department of Hand and Foot Surgery, The Second Affiliated Hospital of Inner Mongolia Medical University, Huimin District, Hohhot, 010030 Inner Mongolia Autonomous Region China; 3 Cardiac Function Department, Cadre Health Care Center, Inner Mongolia Autonomous Region People’s Hospital, Saihan District, Hohhot, 010020 Inner Mongolia Autonomous Region China; 4 Department of Arthroscopy and Sports Medicine, The Second Affiliated Hospital of Inner Mongolia Medical University, Huimin District, Hohhot, 010030 Inner Mongolia Autonomous Region China; 5 Department of Spinal Surgery, The Second Affiliated Hospital of Inner Mongolia Medical University, Huimin District, Hohhot, 010030 Inner Mongolia Autonomous Region China

**Keywords:** Patellar dislocation, Medial patellofemoral ligament, MRI, Recurrence

## Abstract

**Purpose:**

Non-surgical treatment of primary patellar dislocation has a high risk of recurrent dislocation; thus, we tried to identify injuries in which sites of the medial patellofemoral ligament (MPFL) were most associated with recurrent dislocation by analyzing relevant original literature in order to provide improved suggestions on early surgical treatment.

**Methods:**

According to the preset retrieval strategy, the original studies were retrieved until January 2020 using MEDLINE, Embase and Cochrane Library. Review Manager 5.3 software was used to summarize and compare the differences of recurrent dislocation of MPFL injuries at different attachments.

**Results:**

Although the incidence of recurrent patellar dislocation at the femoral attachment of MPFL was higher overall (femoral only vs. patellar only vs. combined: 37.6% vs. 32.3% vs. 35.8%), no statistical difference was found among the three groups (femoral only vs. patellar only, RR = 1.32 [95% CI 0.89–1.95]; *P* = 0.17) (femoral only vs. combined, RR = 1.15 [95% CI 0.59–2.22]; *P* = 0.68) (patellar only vs. combined, RR = 0.94 [95% CI 0.69–1.29]; *P* = 0.72). In addition, the sulcus angle of recurrent dislocation group is significantly greater than that in the non-recurrent dislocation group (MD = 3.06 [95% CI 0.42–5.70]; *P* = 0.02).

**Conclusions:**

Based on the pooled data collected from the original studies available, the risk of recurrent patellar dislocation due to damage to the MPFL at different sites did not differ. Additionally, the sulcus angle in the group with recurrent dislocation was considerably higher when comparing with the group without recurrent dislocation, that is, the shallower and flatter of the trochlear groove, the higher the risk of recurrent patellar dislocation.

## Background

Patellar dislocation is a common traumatic disease, with the incidence of primary patellar dislocation in the population being 23–42 per 100,000 per year. The probability of recurrent dislocation in these patients after conservative treatment is 17–66%, and the younger the patient, the higher the probability of recurrence [1–5]. It is believed that patellar dislocation is a multifactorial disease with the combined effects of bone anatomical abnormality and abnormality in the soft tissue structure that maintains the stability of the patellofemoral joint. Common bone anatomical abnormalities include dysplasia of the trochlear groove of the femur, high patella, increased Q Angle, tibial torsion, genu valgus, etc. [6]. The soft tissue structure abnormalities mainly include the injuries of MPFL and medial patellar support band [7].

Numerous studies have compared the clinical effects of primary patellar dislocation surgery and non-surgical treatment, suggesting that the redislocation rate of surgical treatment is relatively low, but no difference lies in the long-term functional results [8–12]. A Cochrane systematic review concluded that although the short-term effect of primary patellar dislocation surgery was better than that of non-surgical treatment, the level of evidence in these studies was low, hence having a high risk of bias [13]. For this reason, there are still many studies supporting non-surgical treatment of primary patellar dislocation [14, 15]. Considering the high risk of recurrent dislocation in non-surgical treatment, it is necessary to identify which factors are most associated with recurrent dislocation and in order to put forward more optimized surgical treatment.

MPFL is an important static soft tissue structure that limits subluxation and dislocation of the lateral patella, especially when the patella does not enter the trochlear groove within 30° of the knee bend [6] and can provide 50–80% medial mechanical stability for the prevention of lateral patellar dislocation [7, 16, 17]. Elias et al. used cross-sectional MRI to divide MPFL injuries into three regions, namely the femoral attachment, the patellar attachment, and combined injury [18]. Some studies suggest that 75–100% MPFL tears are located in the femoral attachment, while others suggest that up to 50% of MPFL tears are located in the patellar attachment or combined injury [18–21]. In view of the previous study results, we question whether non-surgical treatment of MPFL injury provides predictive information for different prognosis and patellar stability. This study included subject-related original studies to systematically evaluate the risk of recurrent dislocation of MPFL injury with different sites caused by primary patellar dislocation by MRI.

## Methods

### Search strategy

This study was designed and implemented with reference to the PRISMA guidelines. The original research was retrieved until January 2020 using MEDLINE, Embase, and the Cochrane Library. English term for (“patellar” OR “patellofemoral”) AND (“dislocation” OR “subluxation” OR “instability”) AND (“recurrence” OR “redislocation”) AND (“Magnetic Resonance Imaging” OR “MRI”). In addition, the corresponding references of the included literature were searched manually to ensure no eligible studies were missed.

### Inclusion and exclusion criteria

Inclusion criteria: (i) population: patients with primary patellar dislocation and receiving non-surgical treatment; (ii) three types of MPFL injury have been compared in the intervention group and the comparison group, that are, femoral attachment, patellar attachment, and combined injury; (iii) result: risk of recurrent dislocation; (iv) experimental design: prospective or retrospective cohort studies or case-control studies; v) full-text available. Exclusion criteria: (i) acute patellar dislocation does not clarify whether it is primary or recurrent dislocation; (ii) overview and systematic evaluation; (iii) studies involving surgical treatment; (iv) studies that did not report the incidence of recurrent dislocation.

### Clinical data extraction

Two members of the research group extracted the following data from each article included in the study: the first author’s name, year of publication, the level of evidence, number of primary patellar dislocation and recurrent dislocation, the average age of patients, gender, and follow-up time. For each study, the number of femoral attachment, patellar attachment, combined injury at both attachments, and the corresponding number of recurrent patellar dislocation were also extracted. In addition, since three studies have evaluated the difference in femoral sulcus angle between the recurrent and non-recurrent dislocation groups, we also extracted the corresponding data.

### Statistical analysis

The Cochrane Review Manager 5.3 software was used for statistical analysis of all data. Relative risk (RR) and 95% confidence intervals were used to compare the Dichotomous variable (comparison of recurrent dislocation of MPFL injury at different sites). Continuous variables (differences in femoral articular sulcus angle between the recurrent and non-recurrent dislocation groups) were compared using mean difference (MD) and 95% confidence intervals, when *P* < 0.05, there was a statistical difference.

Heterogeneity of included studies was assessed using the *Q* and *I*^2^ tests [22]. If the corresponding *P* > 0.05 or *I*^2^ <50%, the heterogeneity of the included study was considered small, and then the fixed effect model was used for data analysis. Instead, the random effects model was adopted for pooled analysis.

## Result

### Selection for inclusion

**Fig. 1:**
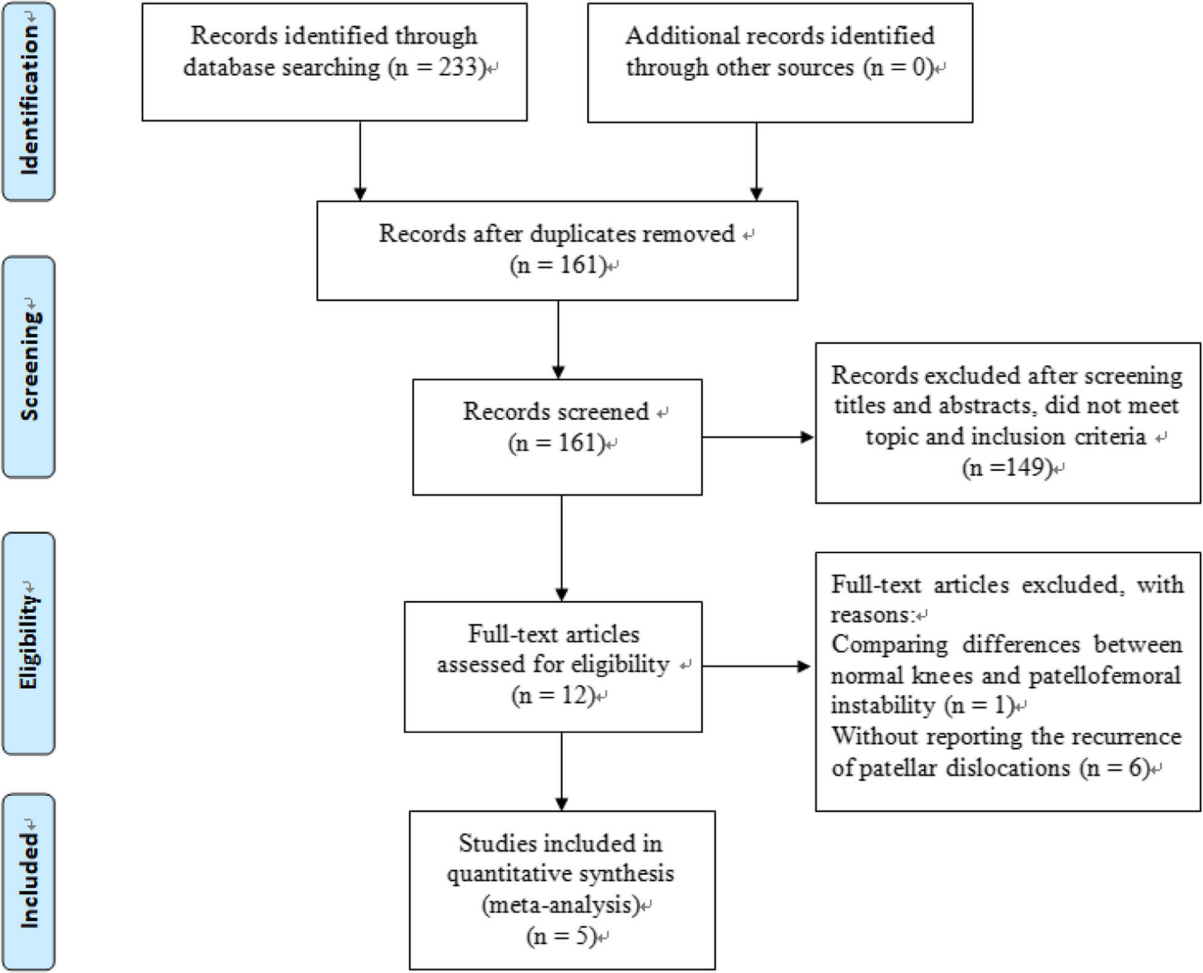
Flow diagram of study selection

Three databases were used to retrieve 233 articles for the first time, and 161 articles were left after removing the duplicate articles. After reading titles and abstracts, 149 papers that did not meet the subject and inclusion criteria were excluded. Among the remaining 12 articles, 1 article compared the difference of MRI between healthy people and patients with patellofemoral joint instability population, 6 article reported cases of MPFL damage in different sites, but did not report the number of recurrent dislocation cases, while last 5 literatures were summarized and analyzed [23–27]. No studies meeting the inclusion criteria were found in the corresponding references of the final included literatures. A PRIMSA flow diagram is presented in Fig. 1.

### Basic clinical characteristics of the included studies

The basic clinical features of the included studies are listed in Table 1. In this study, 2 prospective cohort studies [25, 27], 2 retrospective cohort studies [23, 24], and 1 case-control study were systematically evaluated [26]. The evidence level in all researches is grade III, with their follow-up time being at least 2 years, 4 of which were followed up for an average of more than 5 years. Considering that two of the articles may be continuous studies of different follow-up periods, only the study with a longer follow-up period was included in the data summary analysis [25, 27].

**Table 1:** Study characteristics and patient demographics of the included studies

Author (year)	Study design	LoE	No. of knees	No. of recurrences	Mean age of patients (years)	Gender	Follow-up (years)
Sillanpää 2009 [23]	Retrospective cohort study	III	42	9	20.0 (19–23)	53M	6.9
Seeley 2012 [24]	Retrospective cohort study	III	111	34	14.9 (11–18)	56M:45F	10
Zhang 2018 [25]	Prospective cohort study	III	147	46	20.0 (8–42)	67M:80F	5
Arendt 2018 [26]	Case-control study	III	145	64	17.9	64M:81F	2
Zhang 2019 [27]	Prospective cohort study	III	166	59	18.7 (8–42)	75M:91F	5

*LoE* level of the evidence *M* male, *F* female

### Data summary results

**Fig. 2:**
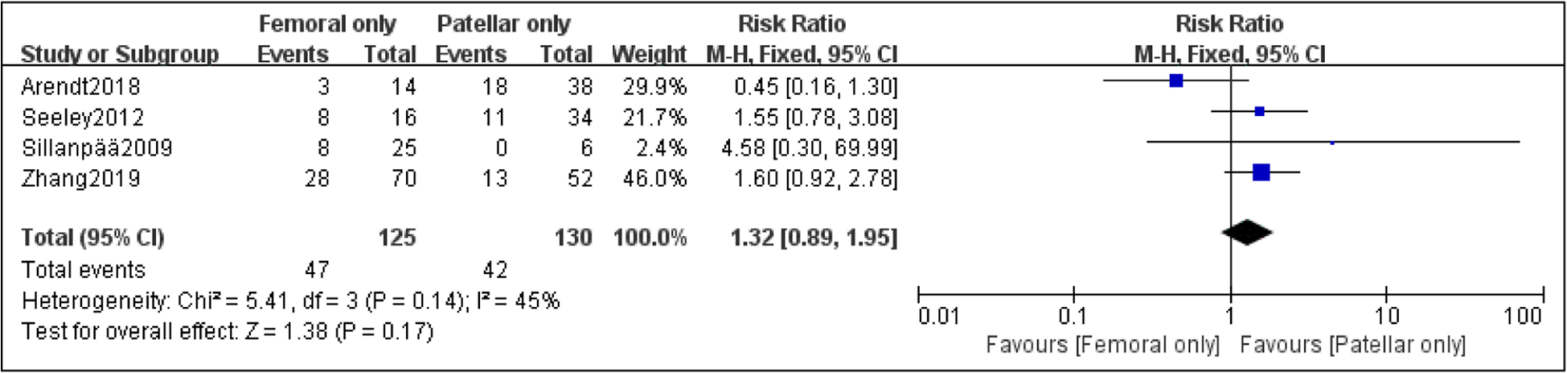
Forest plot of the recurrent dislocation ratio after the injury of MPFL in the femoral attachment and the patellar attachment

**Fig. 3:**
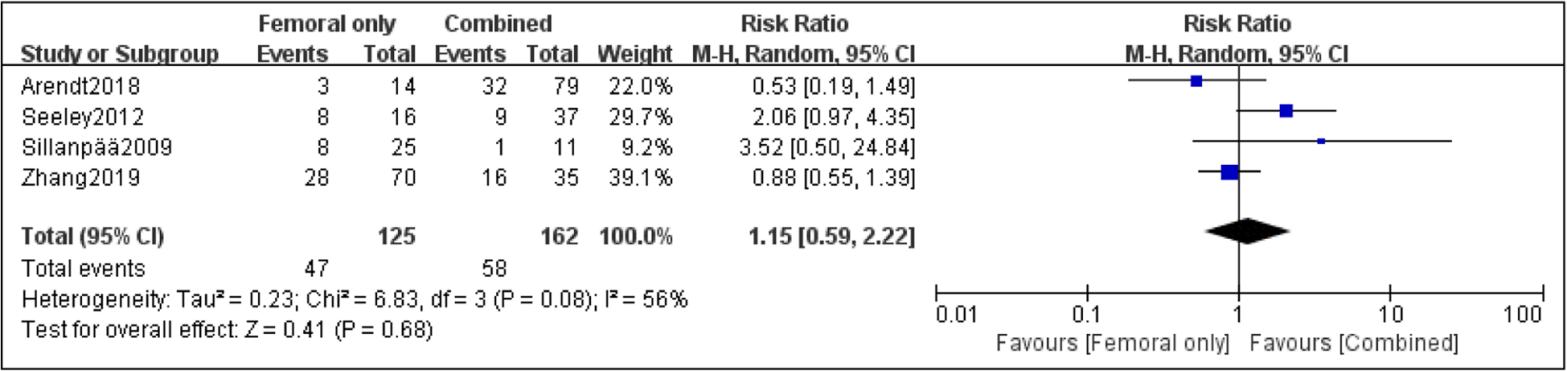
Forest plot of the recurrent dislocation ratio after the injury of MPFL in the femoral attachment and combined injury

**Fig. 4:**
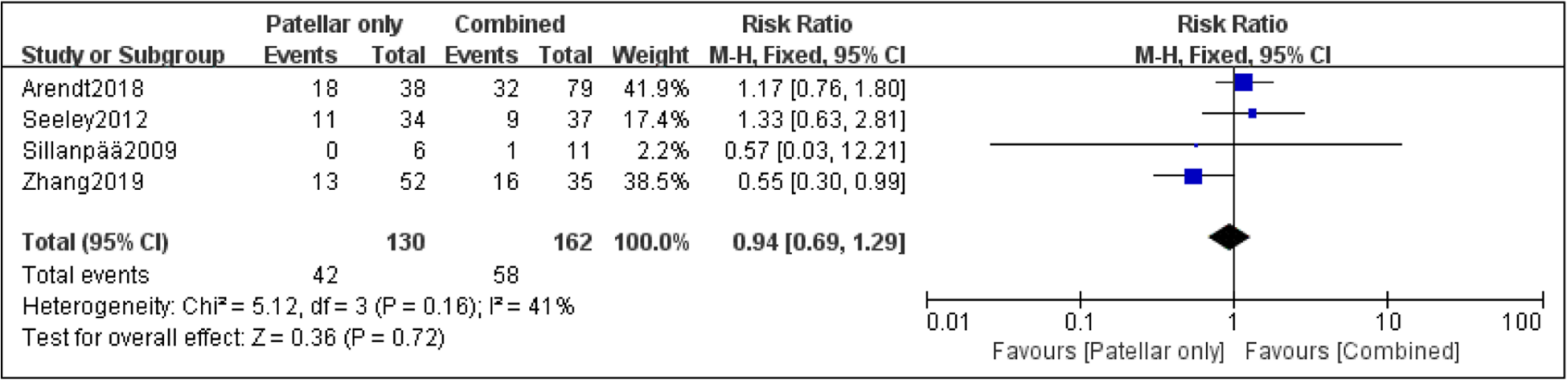
Forest plot of the recurrent dislocation ratio after the injury of MPFL in the patellar attachment and combined injury

**Fig. 5:**
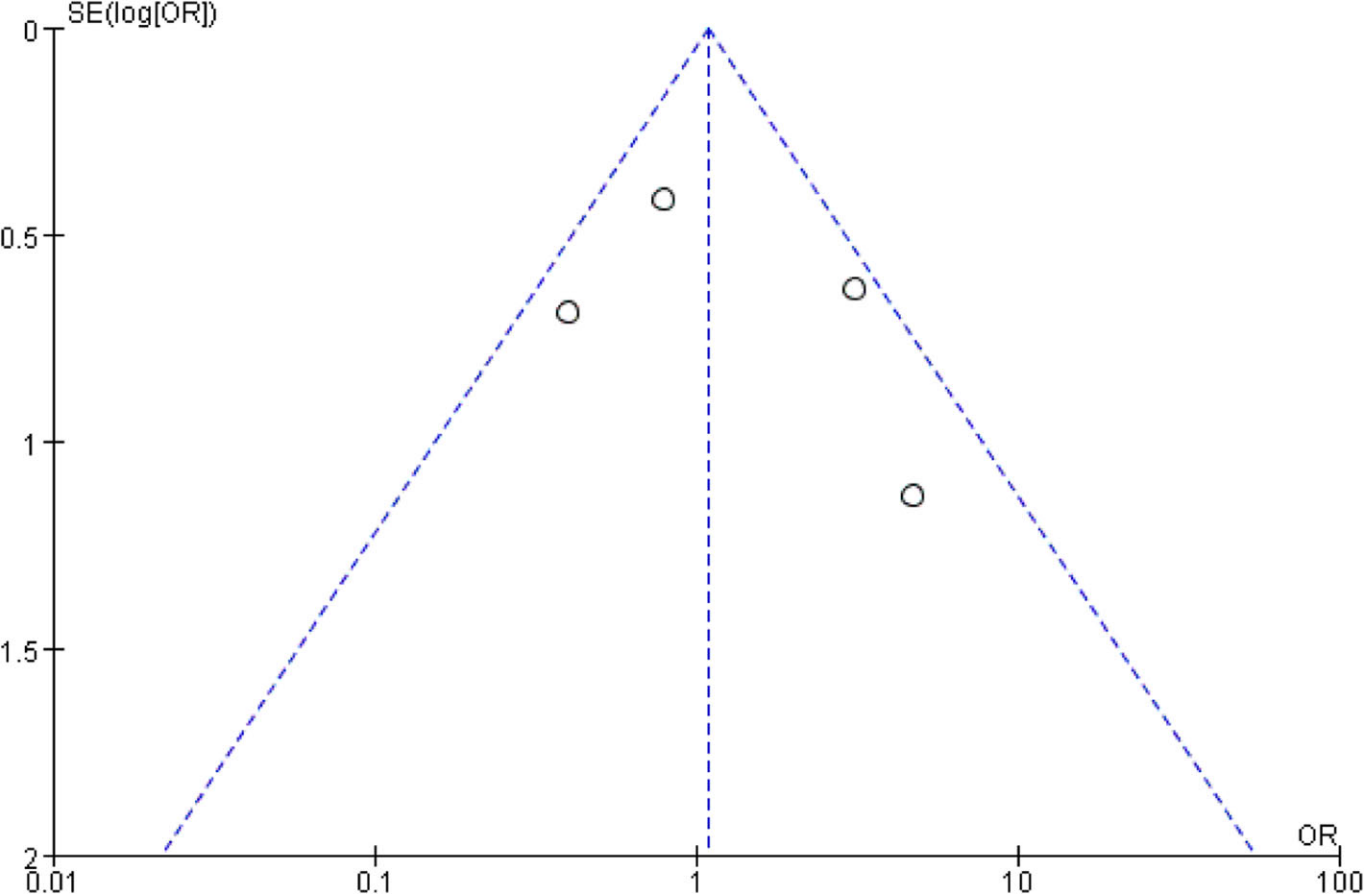
Funnel plot test for publication bias

Four papers were adopted to compare the risk of recurrent dislocation with MPFL injury at different sites [23, 24, 26, 27]. Based on the statistic collected, 125 patients had femoral attachment injury of MPFL, and the incidence of recurrent patellar dislocation was 37.6%; the incidence of recurrent dislocation in 130 patients with patellar attachment injury of MPFL was 32.3%; the incidence of recurrent dislocation in 162 patients with combined injury of MPFL was 35.8%. Although the incidence of recurrent dislocation after the injury at the femoral attachment of MPFL was higher overall, no statistical difference was found among the three groups after data analysis: femoral only vs. patellar only (RR, 1.32 [95% CI 0.89–1.95]; *P* = 0.17) (Fig. 2); femoral only vs. combined, the heterogeneity of this group was higher (*I*^2^ =56%; *P* = 0.08), so the random effect model was used for data summary analysis (RR, 1.15 [95% CI 0.59–2.22]; *P* = 0.68) (Fig. 3); patellar only vs. combined (RR, 0.94 [95% CI 0.69–1.29]; *P* = 0.72) (Fig. 4). No significant publication bias was found by funnel plot test (Fig. 5).

**Fig. 6:**
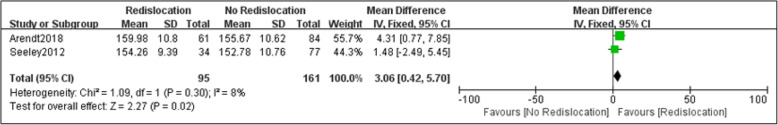
Forest plot of the comparing of sulcus angle between recurrent and non-recurrent dislocation

Three studies were conducted to compare the difference in the angle of trochlear groove between the recurrent and non-recurrent dislocation groups [23, 24, 26], among which Sillanpaa and others researched the difference of femoral subchondral sulcus angle [23], and Seeley and Arendt researched the difference of femoral articular sulcus angle [24, 26]. After conducting summary analysis on the differences in femoral articular sulcus angle, we found that the recurrent dislocation of sulcus angle was significantly greater than non-recurrent dislocation (MD, 3.06 (95% CI 0.42–5.70), *P* = 0.02) (Fig. 6).

## Discussion

This review systematically analyzed the original studies published so far on the correlation between different injuries of MPFL caused by primary patellar dislocation and the risk of dislocation recurrence, trying to find out injuries in which sites of MPFL are more prone to re dislocation. The results showed that no statistically significant difference in the incidence of recurrent patellar dislocation between different MPFL injuries sites (femoral only vs. patellar only, femoral only vs. combined, and patellar only vs. combined). We have also found that the femoral articular sulcus angle in the group with recurrent dislocation was significantly higher than that in the group without recurrent dislocation, indicating that sulcus angle increased, that is, the superficial trochlear groove would increase the risk of recurrent dislocation of the patella.

In this study, the MPFL, as the most important static soft tissue structure limiting lateral patellar dislocation, cannot indicate which injury site has a higher recurrent dislocation risk, which may be related to the small number of samples included in the study. For example, the sample size of Sillanpaa et al. was relatively small, among which only a total of 6 patients had patellar attachment injury, which resulted in no recurrence of patellar dislocation [23]. It is also believed that the probability of MPFL injury is higher in children or adolescents with primary traumatic patellar dislocation, and the injury sites are mostly located at their patellar attachment, while the probability of femoral attachment or combined of MPFL injury in adults is higher than that in children or adolescents [6, 28–32]. In the included study, only one article limited the patient population to adolescents [24], while the other four articles were a combination of adolescents and adults, which might be an important cause of the inapparent difference found among the three groups. In conclusion, based on the current available summary of the study, there was no difference in the risk of recurrent patellar dislocation as a result of the MPFL injuries in different sites.

Dysplasia of femoral trochlear is considered to be an important risk factor for predicting recurrent patellar dislocation. A meta-analysis compared the difference of redislocation between mild and severe trochlear abnormity, based on Dejour’s classification of trochlear dysplasia, and found that the probability of recurrent patellar dislocation in severe trochlear dysplasia was 1.41 times higher than that in mild cases, and the probability of recurrent patellar dislocation in patients with femoral trochlear dysplasia is 4.15 times higher than that in people with normal trochlear development. These results further confirmed the value of femoral trochlear dysplasia in predicting recurrent patellar dislocation [1]. However, the relationship between sulcus angle and recurrent patellar dislocation was not shown in this meta-analysis. On the contrary, we included two original literatures to analyze the difference between femoral articular sulcus angle in the group with recurrent dislocation and the group without recurrent dislocation, and found that sulcus angle in the recurrent dislocation group was significantly higher than that in the non-recurrent dislocation group, that is, the shallower and flatter of the trochlear groove, the higher the risk of recurrent patella dislocation.

The following shortcomings are to be pointed out. First, two of the included studies were retrospective cohort studies, of which one was a case-control study. In the included studies, recall bias, unclear clinical information, uncontrollability of confounders, and other factors might affect the credibility of the pooled data. Secondly, the inconsistencies of clinical variables will also lead to increased heterogeneity of the included studies. For example, the vulnerable sites of MPFL vary from the age, and the longer of the follow-up period, the higher probability of redislocation. Gender differences may also occur, with studies suggesting that women have a higher rate of recurrent patellar dislocation. Hence, the design of future research should focus on improving the above deficiencies, so as to increase the credibility of the research results.

In conclusion, based on the pooled data from the original studies available, the risk of recurrent patellar dislocation did not differ between MPFL injuries at different sites. Additionally, the sulcus angle in the group with recurrent dislocation was considerably higher when comparing with the group without recurrent dislocation, that is, the shallower and flatter of the trochlear groove, the higher the risk of recurrent patella dislocation.

## Data Availability

All data are fully available without restriction.

## Abbreviations

MPFL: Medial patellofemoral ligament
Y: Year
LoE: Level of evidence
M: Male
F: Female
